# Innate immune recognition against SARS-CoV-2

**DOI:** 10.1186/s41232-023-00259-5

**Published:** 2023-01-26

**Authors:** Taisho Yamada, Akinori Takaoka

**Affiliations:** 1 Division of Signaling in Cancer and Immunology, Institute for Genetic Medicine, Hokkaido University, Sapporo, Hokkaido Japan; 2 Molecular Medical Biochemistry Unit, Graduate School of Chemical Sciences and Engineering Hokkaido University, Sapporo, Hokkaido Japan

**Keywords:** SARS-CoV-2, COVID-19, Pattern recognition receptors, Antiviral responses, Interferons, Proinflammatory cytokines, Innate immunity

## Abstract

Severe acute respiratory syndrome coronavirus-2 (SARS-CoV-2) is the causative virus of pandemic acute respiratory disease called coronavirus disease 2019 (COVID-19). Most of the infected individuals have asymptomatic or mild symptoms, but some patients show severe and critical systemic inflammation including tissue damage and multi-organ failures. Immune responses to the pathogen determine clinical course. In general, the activation of innate immune responses is mediated by host pattern-recognition receptors (PRRs) that recognize pathogen-associated molecular patterns (PAMPs) as well as host damage-associated molecular patterns (DAMPs), which results in the activation of the downstream gene induction programs of types I and III interferons (IFNs) and proinflammatory cytokines for inducing antiviral activity. However, the excessive activation of these responses may lead to deleterious inflammation. Here, we review the recent advances in our understanding of innate immune responses to SARS-CoV-2 infection, particularly in terms of innate recognition and the subsequent inflammation underlying COVID-19 immunopathology.

## Background

Coronaviruses, named for the crown-like spikes on their surface, are common pathogens of humans and animals. In the 1960s, the first coronaviruses that infect humans were identified [1]. Four types of human coronaviruses (human coronavirus (HCoV)-229E, HCoV-NL63, HCoV-OC43, and HCoV-HKU1) are prevalent and typically cause some 10 to 15% of common cold symptoms in immunocompetent individuals [2]. SARS-CoV and Middle East respiratory syndrome coronavirus (MERS-CoV), which have confirmed in human in 2002 and 2012, respectively, are highly pathogenic provoking regional and global outbreaks [3–5]. Since December 2019, SARS-CoV-2 has emerged as a major public health crisis, resulting in devastating deaths worldwide [6]. COVID-19 is characterized by both upper and lower respiratory tract infections, which leads to either asymptomatic or a variety of clinical symptoms including cough, fever, and pneumonia, along with other complications like diarrhea and multi-organ failure [7–13]. Risk of severe disease among COVID-19 patients is highly influenced by patients with older age, smoking, chronic obstructive pulmonary disease (COPD), cardiovascular disease, diabetes, obese, cancer, hypertension, and acute kidney injury [14–17].

Recognition of invading viruses by PRRs is the first step of host defense against infection to activate innate immune signaling cascades, which result in the production of types I and III IFNs, proinflammatory cytokines, and chemokines. Types I and III IFNs are critical cytokines to confer cells with anti-viral state [18]. Genetical disruption of these IFNs and the related genes shows higher risk of severe COVID-19 in humans [19–22]. Additionally, a limited and delayed IFN responses in human patients result in exacerbated expression of proinflammatory cytokine, which contribute to severe SARS-CoV-2 symptomatology [23–26]. Indeed, studies of patients with COVID-19 have reported the increment of inflammatory monocytes and neutrophils, a sharp decrease in lymphocytes, and an inflammatory milieu containing interleukin (IL)-1β, IL-6, and tumor necrosis factor (TNF) in severe disease [7, 24, 27–31].

In this review, we review recent findings on the molecular mechanisms of innate immune activation, in particular, by focusing on innate recognition of SARS-CoV-2.

### The life cycle of SARS-CoV-2 in host cells

SARS-CoV-2 is an enveloped virus containing about 30,000 nucleotides of single-stranded, positive-sense genomic RNA [(+)gRNA] complexed with nucleocapsid (N) protein and expresses the viral proteins, spike (S), envelope (E), and membrane (M) structural proteins on its envelope [32, 33]. SARS-CoV-2 primarily infects the nasal and respiratory tract such as nasal epithelial cells, bronchial epithelial cells, alveolar epithelial type II cells, and vascular endothelial cells [34–38]. The S glycoprotein mediates the viral entry into the host cells through its binding of the receptor angiotensin-converting enzyme 2 (ACE2), followed by proteolytic cleavage by host proteases such as transmembrane protease serine 2 (TMPRSS2) at the cell surface or by cathepsin L in the endosome [39–41]. Other host proteins such as neuropilin-1 (NRP1), C-type lectins, furin, KIM-1, and AXL were also identified as cellular cofactors for viral entry [42–46]. In addition, Fc*γ* receptors and CD147 were reported as the other receptors for SARS-CoV-2 infection in monocytes/macrophages and T cells, respectively [31, 47].

Upon viral entry, viral membranes fuse with host membranes to introduce viral (+)gRNA into the cytoplasm [39]. By using the host machineries, the (+)gRNA is translated into large polyproteins (pp1a and pp1ab), which are then cleaved into sixteen types of nonstructural proteins (NSPs) by viral 3C-like protease M^pro^ [48]. Among these NSPs, NSP12 harboring RNA-dependent RNA polymerase (RdRp) catalytic activity and two accessory proteins (NSP7 and NSP8) form a complex [49] and bind to the 3′-untranslated region (3′UTR) of (+)gRNA, which initiate the continuous and discontinuous synthesis of negative-sense RNAs [(−)RNAs] for viral replication and gene expression, respectively [2, 32, 33, 50]. These (−)RNA intermediates serve as a template for the synthesis of (+)gRNA and subgenomic RNAs, and various viral structural proteins and accessory proteins are also translated. Finally, the (+)gRNA is packaged by the structural proteins to assemble progeny virions and bud to release viral particles [6, 33].

In processes of viral entry and replication, virus-derived components such as nucleic acids and the virion proteins are recognized by PRRs on cell surface or in the cytoplasm, leading to the activation of innate responses.

### Sensing of SARS-CoV-2 by PRRs to activate innate immune signaling

The innate immune responses are initiated with the detection of PAMPs or DAMPs by PRRs. Based on the protein domain homology, PRRs can be classified into one of six groups consisted of Toll-like receptors (TLRs), retinoic acid-inducible gene-I (RIG-I)-like receptors (RLRs), C-type lectin receptors (CLRs), nucleotide-binding and oligomerization domain (NOD)-like receptors (NLRs), the absence in melanoma 2 (AIM2)-like receptors (ALRs), and the other types including intracellular DNA sensor, cyclic guanosine monophosphate-adenosine monophosphate (cGAMP), and synthase (cGAS) [18, 51–54]. In this section, we summarize recent reports regarding innate recognition of SARS-CoV-2 and PRR-mediated innate immune signalings during SARS-CoV-2 infection (Table 1).

**Table 1:** Pattern recognition receptors for SARS-CoV-2 infection

Classification	PRR	Ligands	Virus/host*	Experimental models	References
TLR	TLR2	?	–	Computational method with the dataset of BAL^†^ from COVID-19 patients	[55]
		E protein^*a*^	Virus	Mouse BMDM, Human PBMC	[56]
		S protein^*a*^	Virus	Mouse BMDM	[57]
	TLR3	?	–	Genetic variant analysis in patients with life-threatening COVID-19^*b*^	[21]
		?	–	Calu-3/MRC-5 multicellular spheroids	[58]
	TLR4	S protein^*a*^	Virus	Mouse peritoneal macrophages, mouse BMDM	[59]
		S protein	Virus	Mouse peritoneal exudate macrophages, mouse RAW264.7 cells, human THP-1 cells	[60]
		S protein	Virus	In silico study	[61]
	TLR7	?	–	Genetic variant analysis in young men with severe COVID-19	[19]
		?	–	Genetic variant analysis in patients with life-threatening COVID-19^*b*^	[20]
		?	–	Calu-3/MRC-5 multicellular spheroids	[58]
RLR	MDA5	?	–	Calu-3 cells	[62–66]
		(–)RNA	Virus	Calu-3 cells, RIG-I KO A549 cells	[67]
	RIG-I	?	–	Calu-3 cells^*c*^	[66]
		RNA of SARS-CoV-2-infected Vero E6 cells	–	HEK293 cells	[68]
		(+)gRNA	Virus	A549 cells, human primary bronchial, and alveolar epithelial cells (as a direct restraining factor)	[67]
CLR	DC-SIGN L-SIGN LSECtin ASGR1 CLEC10A	S protein	Virus	Human PBMC-derived myeloid cells	[69]
NLR	NLRP3	?	–	Human monocytes derived from COVID-19 patients	[31, 70]
		?	–	Human monocytes in lung tissues of COVID-19 patients	[70]
		?	–	Human PBMC derived from COVID-19 patients	[70]
		?	–	Human monocytes	[70, 71]
		GU-rich RNA	Virus	Human macrophages	[72]
		ORF3a	Virus	HEK293T cells, A549 cells	[73]
		N protein^*d*^	Virus	Mouse BMDM, THP-1 cells, HEK293T cells, A549 cells	[74]
		S protein	Virus	Human macrophages derived from COVID-19 patients	[75]
	NOD1	?	–	Calu-3 cells	[62]
ALR	AIM2	Mitochondrial DNA?	Host	Human monocytes of COVID-19 patients	[31]
Other	cGAS	?	–	ACE2-expressing A549 cells^*e*^	[76]
		DNA?	Host	ACE2-expressing A549 cells, HEK293T cells, HeLa cells^*e*^	[77]
		Mitochondrial DNA	Host	Human endothelial cells in lung-on-chip model	[38]
		Damaged DNA	Host	Human macrophages in skin lesions and lung tissues of COVID-19 patients	[38]

*The origin of each ligand or possible ligand that activates PRRs. †*BAL* bronchoalveolar lavage. The involvement of PRRs and their ligands in SARS-CoV-2 infection is still controversial. In contrast to the supporting evidence regarding usage of some PRRs/ligands as above, there have been contradictory reports that do not support the involvement of some of those PRRs/ligands, although this might be caused by different cell types and/or experimental conditions that were used in each report: ^*a*^Two reports show viral E or S protein do not induce inflammatory response in some types of cells [56, 57]. ^b^There are genetic variant analysis data that do not support the involvement of TLR3 and TLR7 [78]. ^c^As for RIG-I, SARS-CoV-2-induced IFN responses are not affected by RIG-I deficiency in Calu-3 cells [62–67]. ^d^SARS-CoV-2 N protein is reported to inhibit NLRP3-dependent inflammasome in THP-1 cells and human primary monocytes [79]. ^e^Deficiency of STING does not affect types I and III IFN responses in Calu-3 cells during SARS-CoV-2 infection [64, 65]

### TLR-mediated sensing of SARS-CoV-2

TLRs play a crucial role in the activation of innate immune responses against infection with a variety of pathogens [80]. The subcellular localization of TLRs is exclusively in endolysosome or on plasma membrane and generally transduce downstream signalings via two key adaptor molecules, myeloid differentiation factor 88 (MyD88), and Toll/IL-1 receptor domain-containing adaptor inducing IFN-β (TRIF; also known as TICAM-1) [54]. Most of TLRs, except for TLR3, use MyD88 to activate transcription factors, nuclear factor (NF)-κB, and activator protein-1 (AP-1). TLR3 and TLR4 have another adaptor protein TRIF independently of MyD88 to activate NF-κB, AP-1, and IFN regulatory factors (IRFs). TLRs are expressed preferentially in immune cells including monocytes, macrophages, neutrophils, mast cells, basophils, and dendritic cells [81, 82]. In the case of viral infection, numerous studies have shown that in the most case, viral PAMPs for TLRs include viral nucleic acids and proteins. TLR2 is involved in the recognition of viral structural proteins such as EBV-encoded dUTPase, HSV-1-encoded glycoprotein B, and hepatitis B virus capsid [83–85]. TLR4 senses the fusion protein of respiratory syncytial virus (RSV), glycoprotein of Ebola virus, glycoprotein G of vesicular stomatitis virus, and nonstructural protein 1 of dengue virus [86–89]. As for RNA-sensing TLRs, TLR3 has a protective role against SARS-CoV infection in mice [90]. TLR7 is required for type I IFN response during infection with mouse hepatitis virus (MHV), a murine coronavirus, and MERS-CoV in dendritic cells [91, 92].

As for the involvement of TLRs in COVID-19, a single cell-based computational method with the dataset of bronchoalveolar lavage from patients with mild and severe COVID-19 identified TLR2 as a pathogenic factor for the hyperinflammatory response [55]. In this context, it was reported that TLR2 is required for the production of inflammatory cytokines in peripheral blood mononuclear cells (PBMC) during SARS-CoV-2 infection [56]. The extracellular treatment with recombinant SARS-CoV-2 E protein but not S protein induced the proinflammatory cytokine response in bone marrow-derived macrophages (BMDM), and the E protein-triggered response was reduced in TLR2-deficient BMDM [56]. In contrast, there is a report by another research group showing that such an E protein-mediated inflammatory response was not observed in macrophages and lung epithelial cells [57]. In addition, SARS-CoV-2 S protein was shown to be rather immunostimulatory to produce proinflammatory cytokines via TLR2 and TLR4 in macrophages [57, 59, 60, 93]. In silico data indicated that TLR4, TLR6, and TLR1 possess a strong binding affinity to spike protein [61]. Genetic variations in genes encoding TLR3 and TLR7 were shown to be related to the severity of COVID-19 [19–21], while there is also a report showing no significant association [78]. Treatment with TLR3 and TLR7 inhibitors or siRNAs decreased the induction of type I and type III IFNs and proinflammatory cytokines after SARS-CoV-2 infection in Calu-3/MRC-5 multicellular spheroids [58]. Further studies are needed to clarify what PAMPs are directly sensed by TLRs during SARS-CoV-2 infection.

### RLR-mediated sensing of SARS-CoV-2

RLRs such as RIG-I and melanoma differentiation-associated gene 5 (MDA5) are localized in the cytoplasm and recognizes ssRNA and dsRNA, which are virus-derived genomes and replication intermediates and are involved in the recognition of various types of RNA viruses. RIG-I senses RNAs carrying a 5′-triphosphate modification (3pRNA) or short-type dsRNAs in cells infected with a variety of RNA viruses such as influenza A virus, measles virus, and hepatitis C virus, which are widely known to be pathogenic to humans [94–97]. MDA5 mainly recognizes double-stranded RNAs of 3 kb or longer [97] and is required for innate immune responses against certain types of viruses such as Picornaviruses and Flaviviruses [98]. RIG-I and MDA5 consist of two caspase-recruitment domains (CARDs), DExD/H-box helicase domain (HD) and C-terminal domain (CTD). Upon RNA ligand binding via their CTD, the CARDs interact with the adaptor molecule mitochondrial antiviral signaling protein (MAVS; also known as IPS-1, VISA, or Cardif), resulting in gene transcription of types I/III IFNs and inflammatory cytokines [18, 53, 54]. It is also reported that RLRs are required for the sensing of coronaviruses: Both RIG-I and MDA5 are involved in the induction of type I IFNs and proinflammatory cytokines during infection with MHV [56, 99–101]. RIG-I also contributes to the inflammatory cytokine production in response to MERS-CoV infection [102]. However, although it is likely that RLRs sense RNA species derived from such coronaviruses, the detailed mechanism remains poorly understood.

**Fig. 1:**
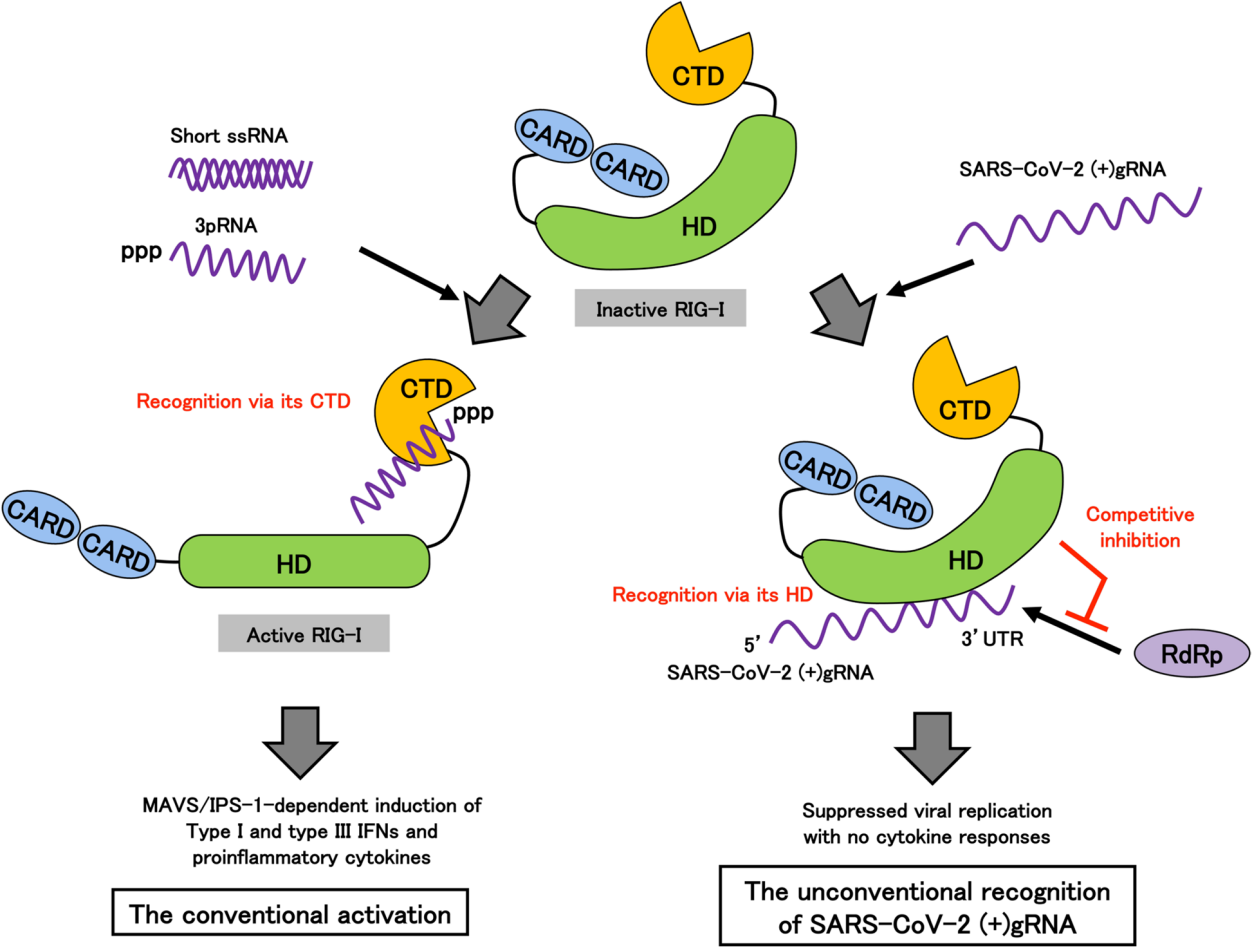
Schematic model of innate recognition of SARS-CoV-2 (+)gRNA. RIG-I consists of two CARDs, HD and CTD. RIG-I conventionally senses 3pRNA or short-type dsRNA via its CTD, followed by the conformational change. And then, oligomerized RIG-I binds to its adaptor protein MAVS/IPS-1, through its CARDs, which resulted in the production of types I and III IFNs and inflammatory cytokines (left). On the other hand, upon SARS-CoV-2 infection, RIG-I preferentially senses the 3′UTR of the viral (+)gRNA through its HD but not CTD. This unconventional recognition of RIG-I fails to activate the downstream MAVS/IPS-1-dependent signaling pathways. Instead, RIG-I directly exerts an antiviral activity via competitive inhibition of the recruitment of viral RdRp to viral (+)gRNA, which blocks the first step of the RdRp-dependent transcription process. CARD, caspase recruitment domain; HD, helicase domain; CTD, C-terminal domain

The basal expression levels of the RLRs are higher in upper airway epithelial cells, macrophages, and dendritic cells of children [103], which may suggest a lower risk for developing COVID-19 in children, compared in those of adults [17]. These data also suggest that RLRs may play a role in the first-line defense against SARS-CoV-2 infection. MDA5 is most likely a candidate intracellular RNA sensor for eliciting innate immune cytokine responses against SARS-CoV-2 in lung epithelial cells. Many studies showed that silencing or knockout of MDA5 but not RIG-I results in reduced types I and III IFN responses during SARS-CoV-2 infection in a lung epithelial cell line, Calu-3 cells [62–67]. However, there is a report showing that RIG-I is also involved in cytokine responses in Calu-3 cells [66]. In addition, transfection with viral RNAs extracted from TMRPSS2-expressing Vero E6 cells infected with SARS-CoV-2 resulted in the induction of innate cytokines in HEK293 cells [68]. In this regard, we confirmed that knockdown of MDA5 but not RIG-I remarkably suppressed the induction of both types I/III IFNs and IL-6 in Calu-3 cells infected with SARS-CoV-2. But we also found that RIG-I is capable to sufficiently restrain SARS-CoV-2 replication in primary human bronchial and alveolar epithelial cells without activation of the conventional RIG-I downstream signaling [67]. The protein expression levels of RIG-I in Calu-3 cells are markedly lower than those of primary human primary bronchial and alveolar epithelial cells as well as A549 cells, which we tested. This may at least partly explain the reason why SARS-CoV-2 replication is observed in Calu-3 cells but not primary human lung epithelial cells. In the first step of viral replication, RIG-I competitively inhibits the access of viral RdRp to the 3′UTR of the viral (+)gRNA through the RIG-I HD. This RIG-I HD-mediated recognition fails to activate the conventional downstream MAVS-dependent IRF/NF-κB signaling pathways, which is in accordance with lack of cytokine induction after SARS-CoV-2 infection in primary human lung epithelial cells (Fig. 1). Consistent with this observation, SARS-CoV-2 can replicate in cells harboring low levels of RIG-I expression such as COPD patient-derived cells, which might link to acute infectious exacerbation in COPD patients [15, 104–106]. Therefore, in the situation where (−)RNA initiates to be transcribed from the viral (+)gRNA, MDA5 in turn play a role as an innate sensor to induce types I/III IFNs and other cytokines. Furthermore, treatment with all-trans retinoic acid (ATRA), which was originally reported to upregulate RIG-I mRNA in a human promyelocytic leukemia cell [107], significantly augments RIG-I protein expression levels in COPD patient-derived bronchial epithelial cells [67]. ATRA or possible other RIG-I inducer(s) may thus be promising agents to enhance preventive and/or therapeutic potentials of COVID-19 patients. RIG-I expression levels are one of the intrinsic determinants for the defense in human lung epithelial cells during the initial process of SARS-CoV-2 infection [67]. Thus, RLRs come into play in a stepwise manner: RIG-I is the first sentinel against SARS-CoV-2 infection in human alveolar and bronchial epithelial cells, and once SARS-CoV-2 initiates to transcribe the (−)RNA, MDA5 functions a major viral sensor to induce innate cytokine responses (Fig. 1).

### CLR-mediated sensing of SARS-CoV-2

CLRs, which are generally expressed in myeloid cells such as dendritic cells, monocytes, macrophages, and neutrophils, are involved in the detection of pathogen-derived mannose, fructose, and glucan carbohydrate structure [108, 109]. Recognition of viruses by CLRs modulates myeloid cell functions including gene transcription, endocytosis, and phagocytosis, thereby regulating antigen presentation, antiviral responses, and T-cell differentiation [109]. Mincle, LSECtin, and CLEC5A act as innate sensors to induce inflammation in response to MERS-CoV, Ebola virus, and dengue virus, respectively [102, 110–112]. On the other hand, some CLRs have been suggested as entry receptors or attachment factors for certain viruses. DC-SIGN promotes viral entry of SARS-CoV, HIV-1, HIV-2, influenza virus, dengue virus, and Ebola virus [113–118]. In addition, L-SIGN and MBL facilitate infection with SARS-CoV and Ebola virus, respectively [119, 120].

A screening assay using ectopic expression for myeloid cell-associated receptors involved in the attachment with SARS-CoV-2 S protein identified five CLRs (DC-SIGN, L-SIGN, LSECtin, ASGR1, and CLEC10A) and Tweety family member 2 (TTYH2) [69]. These five CLRs are expressed by myeloid cells from COVID-19 individuals with hyperinflammation and are engaged in the S protein-mediated robust proinflammatory cytokine response but not actively in virus entry. On the other hand, there are reports showing that the recognition of S protein by DC-SIGN and L-SIGN promotes the entry of SARS-CoV-2 to target cells [121, 122]. Therefore, certain CLRs may play some role in the process of viral entry or the S protein-mediated cytokine responses, although further investigation needs to validate their roles.

### NLR-mediated sensing of SARS-CoV-2

NLRs comprise a large family of intracellular PRRs that are composed of a central NOD and C-terminal leucine-rich repeats and play an important role in the surveillance of the intracellular environment for the presence of infection, noxious substances, and metabolic perturbations [123, 124]. Among NLRs, NLR family, pyrin domain containing 3 (NLRP3) is a well-studied PRR that forms a muti-molecular protein complex, termed inflammasome, upon its activation [125–127]. Its expression is predominantly observed in splenic neutrophils, macrophages, monocytes, and conventional dendritic cells [128]. The activation of NLRP3 inflammasome is a two-step process, priming and activation. The priming step is to upregulate the expression of inflammasome components such as NLRP3, caspase-1, and pro-IL-1β, through the activation of NF-κB upon exposure of inflammatory stimulation such as PAMPs, DAMPs and cytokines. The activation step occurs following the recognition of an NLRP3 activators such as efflux of potassium ion generated by a large array of stimulation including extracellular ATP, microbial agonists, and uric acid crystals. The activated NLRP3 recruits adaptor protein apoptosis-associated speck-like protein containing a CARD (ASC) and caspase-1, which ultimately leads to pyroptosis or the maturation of IL-1β and IL-18 [127]. It is reported that this NLRP3 inflammasome is activated upon infection with viruses such SARS-CoV, encephalomyocarditis virus (EMCV), and influenza virus. In SARS-CoV infection, the NLRP3 inflammasome is mainly activated by viroporins that have ion channel activity [129–131], and that SARS-CoV viroporins enhance viral replication and virulence [132]. Viroporin 2B of EMCV also activates NLRP3 inflammasome via the induction of calcium ion flux [133]. Moreover, the activation of NLRP3 inflammasome is triggered by not only viral protein M2 ion channel but also viral RNA during influenza virus infection [134–137]. NOD1 and NOD2 represent two well-characterized PRRs of the NLR family and recognize conserved motifs of bacterial peptidoglycan, γ-D-glutamyl-meso-diaminopimelic acid (iE-DAP), and muramyl dipeptide (MDP), respectively [138]. After NOD1 and NOD2 sense their ligands, they recruit receptor-interacting serine/threonine-protein kinase 2 (RIPK2), thereby activating the downstream NF-κB and mitogen-activated protein kinase (MAPK) pathways to induce the production of proinflammatory cytokines and antimicrobial responses [138]. Although their roles as bacterial sensors are well-established, several studies uncovered a key function of NOD1 and NOD2 in sensing both RNA and DNA viruses. NOD1 activates type I IFN response in response to *Cytomegalovirus* and hepatitis C virus but not RSV [139–141], whereas NOD2-mediated responses are observed after infection with *Cytomegalovirus*, influenza virus, and RSV [141–143]. These mechanisms of how NOD1 and NOD2 rely on the type I IFN response could be partially explained by their RNA binding properties [140, 141, 144], but it remains still largely unknown.

Elevated proinflammatory cytokines such as IL-1β and IL-18 are characteristic of patients with severe COVID-19 [24]. Several studies particularly focused on the role of NLRP3 in SARS-CoV-2 infection. The concentrations of inflammasome-related markers, IL-1β, IL-18, gasdermin D, and lactate dehydrogenase, were significantly elevated in the serum and plasma of patients with COVID-19, compared with that of healthy donors [31, 70]. In addition, the formation of NLRP3-ASC puncta was detected in monocytes derived from COVID-19 patients. The immunohistochemical staining with lung autopsies of COVID-19 patients confirmed that such puncta were observed in lung tissue-resident monocytes and macrophages [31, 70]. In vitro infection assay also showed SARS-CoV-2 engaged the NLRP3 inflammasome in human monocytes [70, 71]. These results indicated that the NLRP3 inflammasome forms in monocytes and macrophages of COVID-19 patients. Viral RNAs or proteins are proposed as PAMPs responsible for the activation of NLRP3. Treatment with GU-rich single-stranded RNA of SARS-CoV-2 sequence resulted in NLRP3-dependent production of IL-1β in human primary macrophages [72], although the detail mechanism needs further investigation. Open reading frame 3a (ORF3a), a viroporin, and N protein of SARS-CoV-2 triggered IL-1β production in an NLRP3-dependent manner [73, 74]. However, there is an inconsistent report showing that the N protein inhibits the gasdermin D cleavage and IL-1β production in THP-1 cells and human primary monocytes [79]. The stimulation with recombinant SARS-CoV-2 S protein induces NLRP3 activation in macrophages derived from COVID-19 patients but not healthy individuals [75]. In this respect, the authors identified that TLR2 is also required possibly as the priming step for this NLRP3-dependent activation. In support of this, an independent study showed that NLRP3 and IL-1β mRNAs were induced by the treatment with recombinant SARS-CoV-2 E protein in a TLR2-dependent manner, in PBMC and BMDM [56]. Furthermore, TLR8 likely mediates the induction of NLRP3 expression through the detection of GU-rich SARS-CoV-2 sequence RNA [72]. Overall, the NLRP3 inflammasome is activated by various viral PAMPs, and its priming step is coordinated at least by TLRs during SARS-CoV-2 infection. IAnother NLR, NOD1, was identified as a positive regulator for IFN-β mRNA induction in Calu-3 cells during SARS-CoV-2 infection by siRNA-based screening [62], although the detail mechanism is still unclear. Considering that NOD1 modulates MDA5-MAVS complex formation [144], NOD1 may confer an enhancing effect of MDA5-mediated IFN response against SARS-CoV-2 infection.

### ALR-mediated sensing of SARS-CoV-2

ALRs are intracellular innate immune sensors responsible for the detection of DNA and comprise several members of the PYHIN family including AIM2 and IFN-γ-inducible protein 16 (IFI16) [145–147]. AIM2 recognizes cytosolic DNA via its hematopoietic IFN-inducible nuclear protein (HIN) domain, thus inducing the recruitment of the adaptor protein ASC in monocytes and macrophages [148, 149]. This ASC recruitment allows for the formation of a large multi-protein complex, which mediates caspase-1 activation and the maturation of IL-1β and IL-18. The roles of AIM2 were well-established during infection with bacterial pathogens and DNA viruses such as herpes simplex virus-1 (HSV-1), *Cytomegalovirus*, and *Vaccinia virus* [148, 150–152]. On the other hand, in the case of RNA virus infection such as influenza virus, it is reported that AIM2 inflammasome activation is triggered through the accumulation of the oxidized mitochondrial DNA in the cytosol [153]. Based on these findings, both DNA and RNA viruses are likely to activate AIM2 inflammasome.

It is shown that the AIM2 inflammasome is activated in monocytes from patient with COVID-19 [31]. As mentioned above, the expression of inflammatory cytokines including IL-18 is actually upregulated in serum and plasma from severe COVID-19 patients [24, 31, 70]. Analysis with confocal microscopy confirmed ASC specks co-localized with AIM2 in COVID-19 monocytes. It is suggested that AIM2 may sense DNA released from mitochondria [154]; however, further detailed analysis is needed to understand how AIM2 is activated during SARS-CoV-2 infection. In addition, the AIM2-ASC specks also co-localizes with NLRP3, suggesting AIM2 and NLRP3 make the same inflammasome. It would be interesting to investigate the relevance of AIM2 to NLRP3 activation during SARS-CoV-2 infection.

### cGAS-mediated activation of innate signaling by SARS-CoV-2 infection

cGAS is an essential cytosolic DNA sensor to trigger the innate immune responses against microbial infections. Following the binding of dsDNA, cGAS catalyzes the synthesis of a second messenger, cGAMP, in the presence of GTP and ATP, which subsequently binds to activate the adaptor molecule stimulator of interferon genes (STING) [155–157]. And then, STING recruits and activates TANK-binding kinase 1 (TBK1) as well as NF-κB and IRFs, to induce the production of types I/III IFNs and proinflammatory cytokines. In addition to the sensing of exogenous DNA, cGAS can also be activated by endogenous DNA, including DNA released from mitochondria and extranuclear chromatin damaged by genotoxic stress, in autoinflammatory disorders, inflammation, cellular senescence, cancer, and DNA damage response [158–170]. Of note, it is demonstrated that the release of mitochondrial DNA to cytosol is observed during infection with dengue virus, one of *Flaviviruses*, resulting the activation of cGAS [171]. In this context, cGAS has shown a striking antiviral property against not only DNA viruses such as HSV-1, *Vaccinia virus*, and *Cytomegalovirus* but also positive-strand ssRNA viruses including members of Flaviviruses [171–174]. Thus, cGAS activates the downstream signaling through the sensing of viral DNAs or the indirect effects of virus infection that causes mis-localization of self-DNA.

For SARS-CoV-2 infection, lung epithelial cells are the primary site of infection. Experiments with STING-deficient Calu-3 cells suggest that STING-mediated signaling pathway is largely dispensable for types I and III IFN response and the control of viral replication during SARS-CoV-2 infection [64, 65]. However, subsequent studies showed that knockdown of cGAS reduced the mRNA induction of TNF and IL-6 in response to SARS-CoV-2 infection in ACE2-expressing A549 cells [76]. Importantly, in this case, cGAS-STING pathway drives NF-κB-dependent proinflammatory cytokine induction but fails to produce substantial amounts of IFNs, which may partly explain the shift toward an aberrant proinflammatory response [175]. Additionally, SARS-CoV-2 S protein-induced cell fusion causes DNA damage response and induces the formation of micronuclei that are sensed by cGAS, resulting in the induction of IFN-β in ACE2-expressing A549 cells, HEK293T cells, and HeLa cells [77]. Further studies with human primary lung epithelial cells and in vivo models will be required to clarify the anti-SARS-CoV-2 role of cGAS in lung epithelial cells. Besides lung epithelial cells, Domizio D. J. et al. showed that cGAS-STING pathway-driven type I IFN signature is mediated by macrophages adjacent to the areas of endothelial cell damage [38, 175, 176]. Endothelial cells containing damaged mitochondria were detected in skin biopsies and lung tissues from patients with COVID-19. In particular, increased cGAMP levels and phosphorylated STING (STING activation marker) were observed in perivascular macrophages in the skin lesions of COVID-19 patients. Additionally, endothelial cells showed STING-dependent type I IFN response and cell death during SARS-CoV-2 infection. These observations likely explain the mechanisms underlying the aberrant immunopathology at least during the late phase of SARS-CoV-2 infection [38, 175, 176].

### A stepwise model of innate sensor-mediated sensing of PAMPs and DAMPs during SARS-CoV-2

PRR-mediated recognition of invading viruses is the first step of host defense, activating antiviral and inflammatory responses. However, the excessive activation leads to deleterious systemic inflammation. While most individuals infected with SARS-CoV-2 show asymptomatic or mild symptoms, some patients experience severe disease with aberrant inflammation including tissue damage and multi-organ failures. The wide spectrum of clinical manifestation of COVID-19 patients suggests that individual immune responses to SARS-CoV-2 may critically determine the clinical course. Here, we will discuss how PAMPs and DAMPs are spatiotemporally sensed by an array of PRRs in certain cell types, particularly in terms of innate activation.

**Fig. 2:**
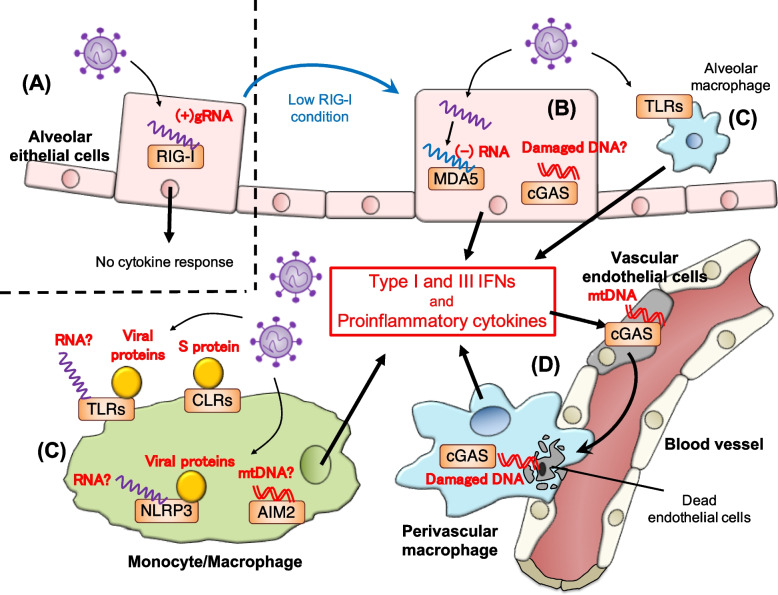
Stepwise model of PRR-mediated activation of innate immune response in lung tissues during SARS-CoV-2 infection. In the first step, RIG-I senses (+)gRNA of SARS-CoV-2 that is released from a viral particle and directly inhibits viral replication without activation of the conventional RIG-I downstream signaling **A**. In the condition of reduced RIG-I expression or in the epithelial cells with low/no levels of RIG-I expression, viral (−)RNAs initiate to be transcribed from (+)gRNA by viral RdRp. Then, MDA5 in turn plays a role as an innate sensor to induce the expression of types I/III IFNs and proinflammatory cytokines. This signaling might result in cell damage, leading to activation of cGAS pathway via possibly the sensing of host nuclear or mitochondrial DNA **B**. In immune cells such as macrophages/monocytes, TLRs, CLRs, NLRP3, and AIM2 function as major innate sensors to recognize viral proteins, nucleic acids, and host DAMPs, resulting in innate cytokine responses **C**. These innate inflammatory responses induce the death of neighboring endothelial cells, leading to the activation of cGAS pathway via its mitochondrial DNA in the cells. The dead endothelial cells are engulfed by perivascular macrophages, and then the host damaged DNA activates cGAS pathway, which induces type I IFNs and proinflammatory cytokines **D**. This cGAS machinery may initiate a self-perpetuating loop of the sterile inflammation, causing the detrimental inflammation in late phase of infection

It has been proposed that SARS-CoV-2 infection primarily targets the respiratory tract [37]. In bronchial and alveolar epithelial cells infected with SARS-CoV-2, RIG-I first senses (+)gRNA of SARS-CoV-2 and acts as a direct restraining factor without the production of types I/III IFNs and proinflammatory cytokines, in an unconventional manner. This may link to asymptomatic (or mild) manifestation of COVID-19 patients. In this respect, the balance between RIG-I expression levels and the load of invading viruses would regulate the fate of viral replication (Fig. 2A), especially in the early phase of infection. If RIG-I could not recognize the (+)gRNA in some cells with downregulated RIG-I expression such as Calu-3 cells and COPD patient-derived lung epithelial cells, MDA5 in turn senses viral (−)RNA transcribed from the (+)gRNA by viral RdRp. Also, cGAS may sense host damaged DNA as a DAMP in the infected epithelial cells. Thus, MDA5 and cGAS mainly contribute to the production of types I/III IFNs and proinflammatory cytokines in epithelial cells in the early phase of infection (Fig. 2B). On the other hand, a variety of PRRs such as TLRs, CLRs, NLRs, and AIM2 come into play for the recognition of viral PAMPs as well as host DAMPs, particularly in macrophages and monocytes, which may likely lead to exacerbated proinflammatory cytokine production with extensive infiltrations of inflammatory cells in the respiratory tract in the late phase (Fig. 2C). Such multiple innate sensor-mediated inflammation disrupts mitochondrial homeostasis of vascular endothelium, resulting in the cytosolic accumulation of mitochondrial DNA, which activates cGAS-STING pathway for inducing innate cytokine responses and cell death. The dead vascular endothelial cells are engulfed by macrophages at the perivascular lesions, which also lead to cGAS activation. This innate sensor-mediated signaling circuit may contribute to sustained, dysregulated cytokine responses (Fig. 2D), which may explain COVID-19 immunopathology at least in the late phage of infection.

In summary, we would propose that PRR-mediated innate antiviral defense during SARS-CoV-2 infection consists of at least three steps as follows: (1) RIG-I-mediated direct antiviral responses cell without cytokine response, (2) PAMP-dominant activation of innate signaling with cytokine response, and (3) DAMP-dominant activation of innate signaling with dysregulated cytokine response. While innate immune cytokines play a beneficial role for the successful clearance of invading viruses in the early stage, their excessive production in the late stage may contribute to aggravate COVID-19 immunopathology.

## Conclusions

Not only nucleic acid sensors but also other types of PRRs have been reported to be involved in the activation of innate cytokine responses against SARS-CoV-2. In the early phase of SARS-CoV-2 infection, both types I and III IFNs can exert their antiviral activities. In this respect, it is also reported that types I and III IFNs are important cytokines to inhibit viral infection by inducing antiviral genes including anti-SARS-CoV-2 genes such as LY6E and BST2 [177, 178]. However, severe COVID-19 patients fail to suppress viral replication in the early phase of infection due to insufficient and delayed types I and III IFN responses, which results in exacerbated proinflammatory cytokine production in the late phase [24–26]. Several clinical trials showed that early administration of types I and III IFNs significantly prevented the clinical deterioration and reduced the duration of detectable virus [179–182], whereas later administration of type I IFNs was associated with increased mortality [179, 180], highlighting the opposing effects of type I IFNs for host protection and immunopathology. This harmful effect may be partially explained by a recent report showing that both types I and III IFNs disrupt lung epithelial repair during recovery from viral infection [183]. On the other hand, circulating neutralizing autoantibodies against type I IFNs are found in about 10% of patients with critical COVID-19 and in elderly individuals but not in young individuals with asymptomatic or mild SARS-CoV-2 infection [184–186], suggesting the generation of autoantibodies against type I IFNs may contribute to the pathogenesis of severe COVID-19 in the late phase of infection. It would be speculated that in addition to type I IFNs, some of other cytokines such as IL-6 might also have an opposing effect in early and late phases of SARS-CoV-2 infection. Consistently, it was reported that IL-6 production levels in the early phase of RSV infection are correlated with the limitation of disease severity through its effect on maturation of regulatory T cells [187], whereas high concentrations of IL-6 in the late phase of infection correlate with respiratory failure, ARDS, and adverse clinical outcomes [188]. Thus, better understanding of molecular mechanisms underlying innate recognition-mediated immune responses in terms of immunopathology will aid to provide a therapeutic design of cytokine or anti-cytokine strategy with optimal timing and duration.

## Data Availability

Not applicable.

## Abbreviations

ACE2: Angiotensin-converting enzyme 2
AIM2: Absence in melanoma 2
ALR: AIM2-like receptors
AP-1: Activator protein-1
ASC: Apoptosis-associated speck-like protein containing a CARD
ASGR1: Asialoglycoprotein receptor 1
ATRA: All-trans retinoic acid
AXL: Tyrosine-protein kinase receptor UFO
BMDM: Bone marrow-derived macrophage
BST2: Bone marrow stromal cell antigen 2
CARD: Caspase-recruitment domain
cGAMP: Cyclic guanosine monophosphate-adenosine monophosphate
cGAS: cGAMP synthase
CLEC5A: C-type lectin domain containing 5A
CLEC10A: C-type lectin domain containing 10A
CLR: C-type lectin receptor
COPD: Chronic obstructive pulmonary disease
COVID-19: Coronavirus disease 2019
CTD: C-terminal domain
DAMP: Damage-associated molecular pattern
DC-SIGN: Dendritic cell-specific intracellular adhesion molecule-3-grabbing nonintegrin
E: Envelop
EMCV: Encephalomyocarditis virus
HCoV: Human coronavirus
HD: Helicase domain
HIN: Hematopoietic IFN-inducible nuclear protein
HIV: Human immunodeficiency virus
HSV-1: Herpes simplex virus-1
iE-DAP: *γ*-D-Glutamyl-meso-diaminopimelic acid
IFI16: IFN-*γ* inducible protein 16
IFN: Interferon
IL: Interleukin
IRF: IFN regulatory factors
KIM-1: Kidney injury molecule-1
LSECtin: Liver and lymph node sinusoidal endothelial cell C-type lectin
L-SIGN: Liver and lymph node-specific intracellular adhesion molecule-3-grabbing nonintegrin
LY6E: Lymphocyte antigen 6 family member E
M: Membrane
MAPK: Mitogen-activated protein kinase
MAVS: Mitochondrial antiviral signaling protein
MDA5: Melanoma differentiation-associated gene 5
MDP: Muramyl dipeptide
MERS-CoV: Middle East respiratory syndrome coronavirus
MHV: Mouse hepatitis virus
MyD88: Myeloid differentiation factor 88
N: Nucleocapsid
NF-кB: Nuclear factor-кB
NLR: NOD-like receptor
NLRP3: NLR family, pyrin domain containing 3
NOD: Nucleotide-binding and oligomerization domain
NRP1: Neuropilin-1
NSP: Nonstructural protein
ORF3a: Open reading frame 3a
PAMP: Pathogen-associated molecular pattern
PBMC: Peripheral blood mononuclear cell
RdRp: RNA-dependent RNA polymerase
PRR: Pattern-recognition receptor
RIG-I: Retinoic acid-inducible gene-1
RIPK2: Recruit receptor-interacting serine/threonine-protein kinase 2
RLR: RIG-I-like receptor
RSV: Respiratory syncytial virus
PYHIN: Pyrin and HIN domain
S: Spike
SARS-CoV-2: Severe acute respiratory syndrome coronavirus-2
STING: Stimulator of IFN genes
TBK1: TANK-binding kinase 1
TLR: Toll-like receptor
TMPRSS2: Transmembrane protease serine 2
TNF: Tumor necrosis factor
TRIF: Toll/IL-1 receptor domain-containing adaptor inducing IFN-*β*
TTYH2: Tweety family member 2
UTR: Untranslated region
3pRNA: RNAs carrying a 5′-triphosphate modification
(−)gRNA: Negative-sense RNA
(+)gRNA: Positive-sense genomic RNA.
